# A qualitative study of nursing practitioners' experiences with COVID-19 patients dying alone in Greece

**DOI:** 10.3389/fpubh.2022.981780

**Published:** 2022-10-21

**Authors:** Polychronis Voultsos, Anna Tsompanian, Maria Deligianni, Eftychia Tsamadou, Alexandra K. Tsaroucha

**Affiliations:** ^1^Laboratory of Forensic Medicine & Toxicology, School of Medicine, Faculty of Health Sciences, Aristotle University of Thessaloniki, Thessaloniki, Greece; ^2^Postgraduate Program on Bioethics, Laboratory of Bioethics and Laboratory of Experimental Surgery and Surgical Research, School of Medicine, Democritus University of Thrace, Alexandroupolis, Greece

**Keywords:** death in isolation, dying alone, hospitalized COVID-19 patients, COVID-19 pandemic, nursing practitioners/professionals

## Abstract

**Background:**

In Greece, there is still limited research on death in isolation due to COVID-19. This deserves attention because of the recent financial crisis, which profoundly impacted public health, and the high relevance of the Hippocratic tradition to the moral values of clinical practice.

**Methods:**

A prospective qualitative study using in-depth interviews with 15 frontline nursing practitioners working in a COVID-19 ward or intensive care unit (ICU) was conducted from July 2021 to December 2021.

**Results:**

The inability of family members to say a final goodbye before, during, or after death by performing proper mourning rituals is extremely inhuman and profoundly impacts the mental health status of patients, family members, and nursing practitioners. Patients and their family members strongly desire to see each other. Epidemiology, liability, and proper nursing performance emerged as reasons for the enforced strict visitation restrictions. Participants emphasized that visitations should be allowed on an individual basis and highlighted the need for the effective use of remote communication technology, which, however, does not substitute for in-person contact. Importantly, physicians allowed “clandestine” visits on an individual basis. Nursing practitioners had a strong empathic attitude toward both patients and their families, and a strong willingness to provide holistic care and pay respect to dead bodies. However, they also experienced moral distress. Witnessing heartbreaking scenes with patients and/or their families causes nursing practitioners to experience intense psychological distress, which affects their family life rather than nursing performance. Ultimately, there was a shift from a patient-centered care model to a population-centered care model. Furthermore, we identified a range of policy- and culture-related factors that exaggerate the negative consequences of dying alone of COVID-19.

**Conclusion:**

These results reinforce the existing literature on several fronts. However, we identified some nuances related to political decisions and, most importantly, convictions that are deeply rooted in Greek culture. These findings are of great importance in planning tailored interventions to mitigate the problem of interest and have implications for other similar national contexts.

## Introduction

Death and end-of-life care have radically altered during the COVID-19 pandemic. Due to strict “no-visitor rules,” which aim to prevent the virus from spreading, COVID patients are hospitalized in isolation and die in isolation. Family members are prohibited from saying last goodbyes to their loved ones (before, during, or after death through mourning rituals).

In-person contact is a hallmark of high-quality end-of-life care (1). Of the many unprecedented adverse outcomes of the COVID-19 disease, one “stands out as particularly vile, the experience of dying alone” (2). COVID-19 patients are dying “alone, surrounded by machines and strangers wearing heavy protective gear” (2), namely, a death that Callahan calls “wild death” (3). Too often patients die without being surrounded by or communicating with their family members. Remote communication strategies can promote effective face-to-face communication between dying patients and their families, which is important for family members (4). Nevertheless, it is argued that even if this connection did take place, family members felt like they did not get to say final goodbye “properly” to their loved ones (5). Such situations may have serious (presently unknown) psychological consequences for family members and healthcare providers, such as feelings of helplessness, frustration, guilt, moral distress, and prolonged complicated grief (6). In addition, the pandemic has changed funerals and significantly increased the risk of complicated grief (7, 8). Jordan et al. recommended a three-level public health approach to managing surges in complicated grief (8). Dying a lonely death may lead to post-traumatic stress and cause long-term negative consequences for grieving relatives (9). It is true that the circumstances surrounding the hospital stay of patients in COVID-19 wards or intensive care units (ICUs) involve conditions such as “contemporary” dying in isolation for which we are unprepared as a society (10).

A systematic review and meta-analysis conducted by Pappa et al. highlighted the high prevalence rates of depression, anxiety, and insomnia among frontline healthcare professionals during the COVID-19 pandemic (11). It has been argued that physical and social rewards and support are crucial to maintaining the mental health of nursing practitioners (12), who are experiencing a shocking transition from patient-centered care to population-based care, which goes against their basic training and core values and beliefs in the current epidemiological context (13).

In addition, it is of great importance that the phenomenon of patients dying alone of COVID-19 may give rise to major occupational health problems. Providing care for and being in close relationship with COVID-19 patients dying in isolation is a perceived traumatic and highly stressful event. As such, it may contribute to burnout among nursing practitioners, which remains one of the major occupational health problems. Below, we provide more details on burnout among nursing practitioners.

Burnout syndrome (BOS) is a psychological syndrome resulting from poor management of chronic workplace stress (14–16). Burnout syndrome has three dimensions: it is characterized by emotional exhaustion (EE, mental and generalized fatigue), depersonalization (DP, cynicism represented by negative feelings and detached attitude toward patient care), and reduced personal accomplishment (PA, poor professional self-esteem/self-conception/efficacy) (14–17). EE is the core component of this syndrome (17). Burnout syndrome was recently included in the 11th version of the International Classification of Diseases (ICS-11) (15).

Burnout syndrome is a “recognized and well-established workplace hazard in the healthcare sector,” and has become an important public health problem (16, 18). Chirico et al. conducted an interesting umbrella review of systematic reviews and meta-analyses concerning the prevalence of burnout syndrome in healthcare workers and found that low personal achievement and high EE had the highest prevalence among nursing practitioners (16), especially among frontline nursing practitioners working in emergency departments (16, 19–21).

As nursing practitioners work in very stressful environments and often have limited resources and excessive workloads, they are particularly prone to developing burnout syndrome. Intensive care unit (ICU) nursing practitioners working in extremely stressful (involving high emotional stress) and demanding environments are at a higher risk of developing burnout syndrome. Indeed, nursing practitioners “often have to deal with end-of-life matters, continuous suffering of patients, demands and distress of relatives, and, sometimes, ethical issues” (17). In that regard, overwhelming scientific evidence indicates that ICU nursing practitioners have a higher prevalence of burnout compared to nursing practitioners working in other units (17).

During the COVID-19 pandemic (especially during the first wave), the risk of burnout in nursing practitioners working in COVID-19 wards or ICUs increased considerably (16, 17). Tarchi et al. state that in a study “healthcare workers exhibited a higher risk of experiencing anxious or depressive symptoms after the onset of COVID-19, as well as a higher risk of burnout” (22). In a study conducted in Belgium during the first wave of the pandemic, 68% of ICU nursing practitioners were found to be at high risk of burnout. Regarding the three dimensions of burnout, the risk of emotional exhaustion (EE) was 38%, reduced personal accomplishment (PA) was 31%, and depersonalization (DP) was 38, 31, and 29%, respectively. These rates were apparently higher than the corresponding rates mentioned in other studies conducted in Belgium before the COVID-19 pandemic in ICU nursing practitioners or a general nursing population (17).

Nursing practitioners with high empathy are prone to develop close and longstanding emotional bonds with COVID-19 patients. These bonds may lead nursing practitioners to feel a deep sense of grief over a patient's death or dying process (23). The deep sense of grief that frontline nursing practitioners feel toward dying COVID-19 patients may put them at risk of burnout syndrome. Grief may be an overlooked predisposing factor for the development of burnout in frontline nursing practitioners, which mainly affects the dimension of depersonalization (DP) and only marginally affects the dimension of emotional exhaustion (EE) (24, 25). Bruyneel et al. showed a positive correlation between the number of deaths among COVID-19 patients and the risk of low personal accomplishment (17). Furthermore, Boerner et al. argued that high supervisor (not coworker) support and caregiving benefits may play a protective role in the development of burnout due to the build-up of grief over patient death. Moreover, it is argued that when coping strategies are effective to overcome stress burnout is less likely to happen (26). Lack of individual coping strategies are predictive of burnout syndrome (16). Boerner et al. stated that enhancing nursing practitioners' ability to “manage their emotions related to patient death may be more effective than trying to prevent grief or the relationships that cause grief” (25). To that effect, it is to be noted that the interaction between coping mechanisms and personal factors in the development of burnout has not yet been fully elucidated (22).

Nevertheless, while empathy-driven grief may lead to the development of burnout among nursing practitioners, it is mentioned that “high levels of empathy can be protective against the development of burnout and, on the contrary, when burnout is present it might not permit a fully empathic therapeutic relationship with patients” (26). Ferri et al. found that low levels of empathy could make people more vulnerable to burnout (26). In that regard, it is to be noted that Tarchi et al. found that emotional stability is associated with lower risk of anxiety and depression but also may be a predisposing factor for depersonalization (22).

Among the different job demands and resources that are reported as significant predictors of burnout in healthcare workers during the COVID-19 pandemic include work characteristics and working conditions (e.g., high perceived workload/job demands, irregular shift schedules, close and longstanding relationships with patients and their relatives who often put nursing practitioners under pressure), interpersonal relationships (e.g., bad/insufficient communication with colleagues), role conflict and emotional demands, shortage of healthcare resources, lack of personal protective equipment, and, ultimately, and most importantly, numerous deaths of COVID-19 patients, which are associated with a high risk of low personal accomplishment (PA) (16, 17, 27, 28). Work-related factors, such as exposure to traumatic events, are highlighted as important risk factors for developing burnout in emergency nurses, in whom burnout rates are high (16, 19). Furthermore, Galanis et al. in their study (systematic review and meta-analysis) argue that among the main risk factors of developing burnout syndrome in nursing practitioners working in COVID-19 healthcare settings are the following: “increased perceived threat of Covid-19, longer working time in quarantine areas, working in a high-risk environment, working in hospitals with inadequate and insufficient material and human resources, increased workload and lower level of specialized training regarding COVID-19” (29). It is important to note that nursing practitioners' exposure to the traumatic event of caring for patients dying in isolation has received little attention as a predisposing factor for the development of burnout syndrome in nursing practitioners.

Moreover, it has been reported that a shortage of personal protective equipment increases the risk of burnout (especially of the dimension EE, which is the core component of the syndrome), obviously because of a fear of contracting the virus and transmitting it to others (patients or their loved ones) (17). “A significantly increased risk of anxiety, depression, and sleep disturbances was found in HCWs who had experienced unprotected exposure to patients with COVID-19” (20).

Anxiety, depression, and burnout, seem to be inter-mutually independent albeit divergent reactions to various stressors (22). The interaction between these three constructions has not yet been fully elucidated (22). Researchers should bear in mind that there are associations and overlaps between burnout and depression as individual outcomes. However, these associations cannot be reliably determined due to inconsistencies in the definitions and assessment methods for burnout across studies (16). Anxiety and depression symptoms are included among the factors that are associated with the prevalence of burnout syndrome among healthcare providers (16). Tarch et al. state that literature “showed a directional role for anxious and depressive symptoms in burnout” (22). It is argued that depression is associated with higher levels of emotional exhaustion, while anxiety is associated with lower levels of personal accomplishment (22).

Magnavita et al. conducted an interesting umbrella systematic review and concluded that “it is simplistic to attribute the findings of EE, DP, and LPA to the coronavirus outbreak without delving into the numerous factors that can affect the phenomenon” (20). However, the authors did not focus on nursing practitioners' exposure to patient death as a factor associated with burnout risk.

Burnout syndrome may significantly negatively impact nursing practitioners' mental health state, individual, family, and professional life (e.g., causes bad relationships, “impaired quality of care and patient satisfaction with care, medical errors, and reduced patient satisfaction”) (16). Burnout syndrome in nursing practitioners is associated with adverse health effects and increased turnover (17). In addition, BOS has negative consequences at the organizational level (16).

Hospitals and governmental authorities need to pay specific attention to work-related stress risk factors to prevent or reduce the phenomenon among healthcare workers (16). Psychological interventions for nursing practitioners at the individual, family, or organizational levels should be implemented to improve nursing practitioners' resilience (16).

Greece is a county where the phenomenon of dying alone of COVID-19 deserves to be explored. There are healthcare workforce shortages in the current epidemiological context, which, however, have been happening for years in many other developing and developed countries (30, 31). However, in Greece, there are additional factors that make the problem bigger: (a) The Greek financial crisis has taken a heavy toll on public sector healthcare spending over the last decade. (b) All unvaccinated healthcare professionals were given mandatory unpaid leave from work. (c) Lack of training courses dedicated to the management of patients with severe respiratory impairment related to the COVID-19 disease. As a consequence, healthcare professionals remained unable to improve the use of available resources. Furthermore, Lytras and Tsiodras found that equity and quality of care have received less attention in the current epidemiological context (32).

In Greece, there is limited research on the process of dying in isolation from COVID-19. To fill this knowledge gap, this study was designed to examine nursing practitioners' experiences of caring for critically ill patients dying in isolation. Like many other countries, Greece has been strongly affected by the COVID-19 pandemic and has experienced an unprecedented excess of mortality in hospitals (COVID-19 units). In this study, we attempt to shed further light on the phenomenon of dying from COVID-19 in isolation from the perspective of nursing practitioners working in COVID-19 wards or ICUs.

## Methodological aspects

### Objective

The present study was a prospective, qualitative, in-depth, semi-structured interview conducted with 15 experienced frontline nursing practitioners working in COVID-19 wards.

### Research questions

The primary research question that defined the focus of this study was as follows:

What are the lived experiences of nursing practitioners caring for patients dying alone or nearing death in isolation during the COVID-19 pandemic in Greece?

The secondary research question was as follows:

What are the challenges faced by nursing practitioners striving to foster a culture of “good deaths” in COVID wards?

### Study design

#### Methods

Thematic analysis was selected as the methodological orientation to underpin the study.

#### Ethical framework

Prior to participating in this study, the participants were given adequate information on the aim, procedure, nature and confidentiality of the study and the processing of the data according to the ethics approval received for research involving human participants. Subsequently, the participants were asked to provide their informed consent. The authors confirm that informed written consent was obtained from all subjects. Only subjects who voluntarily provided informed consent were included in the study.

#### Inclusion criteria

Nursing practitioners must have met all of the following inclusion criteria to be eligible for participants in this study: (a) Being working as frontline nursing practitioners in a COVID-19 ward or intensive care unit (ICU), (b) caring for critically ill patients, and (c) since the beginning of the pandemic.

#### Exclusion criteria

Nursing practitioners who (a) dropped out, (b) did not work frontline, or were absent from COVID-19 wards or intensive care units for a long period of time (>3 months), (c) did not understand the purpose of the study, or (d) did not agree to participate in the study were excluded.

#### Participant selection

Purposive sampling was used to identify nursing practitioners who had professional experience with critically ill COVID-19 patients. Potential participants were approached via an invitation letter sent by email by the interviewer's (AT) personal acquaintances. Finally, 15 frontline nursing practitioners were selected from the COVID-19 wards and ICUs in different tertiary Greek hospitals. Snowball sampling was used to increase non-probability.

#### Setting

All interviews were conducted in neutral and quiet places in a comfortable environment of the participant's choice. No one aside from the participant or the interviewer was present during the interviews. The interviewer had explored beforehand her own perspective and was emotionally prepared to be able to control her possible influence on the interview.

#### Description of the sample

A summary of participants' demographic characteristics is provided in Table 1. The participants included in the study (*N* = 15) were nursing practitioners who met the aforementioned inclusion criteria and were working in the capital region of Attica. All but one participant represented a wide range of previous work experience in nursing The years of previous work experience ranged from 17 to 30 years, with only two participants having 2 and 14 years of previous work experience, respectively. Most participants were women of varying ages, with only 2 out of a total of 15 men. Regarding their educational background, all participants had graduated from higher-education nursing schools. Nine of the 15 participants in this study had a master's degree. The mean (SD) previous work experience of the 14 participants was 23.5 (SD = 5), with only one participant having 2 years of previous work experience. All participants were working in tertiary referral hospitals. Most participants (*N* = 13) resided and worked in the capital region of Attica (Athens and the suburbs). Two participants were working in Thessaloniki (Greece's second city) and (the university hospital of) Alexandroupolis, respectively. The participants' characteristics are presented in Table 1.

**Table 1:** Participant characteristics.

**Participant**	**Work experience**	**Gender**
P1	19	Female
P2	23	Female
P3	22	Female
P4	30	Female
P5	26	Female
P6	26	Female
P7	27	Female
P8	28	Male
P9	28	Female
P10	21	Female
P11	17	Female
P12	19	Female
P13	14	Female
P14	30	Male
P15	2	Female

#### Data collection

Interviews were conducted in Greek language, in person (face-to-face) and in line with the governmental COVID-19 safety guidelines. All the current COVID-19 protocols were observed. First, two pilot interviews are conducted. Based on pilot interviews and a review of the relevant literature, an interview guide was developed.

The interviews were semi-structured and started with questions such as “During the COVID-19 pandemic, many hospitalized patients were dying or nearing a lonely death while suffering agony. Can you please describe in detail what this situation means?” (*a grand tour question to make the participant comfortable*), “What is it like to be a nursing practitioner caring for a patient who is dying a lonely death or nearing death in isolation?” “How does caring for a patient who is dying alone or nearing death in isolation affect your professional or private life?” “In your view, what are the reasons behind the strict visiting rules imposed by hospital COVID settings, which leave patients isolated?” “In your opinion, under what circumstances should be adopted exceptions (if any) to the general no-visitor rules?” “How might you facilitate continuing bonds between grieving persons and your patient when the policies do not allow them to visit with their beloved ones before, during, or after death?” “What challenges were faced by you in dealing with patients' close relatives who were not allowed to get in contact with their dying or critically ill loved ones, or when their loved ones' unclothed deceased body was delivered to them in plastic unopenable bags?”. Additional questions were asked to elicit more detailed explanations and to identify the essential themes of nursing practitioners' perceptions of the topic of interest.

The interviews were audio recorded and transcribed verbatim. Field notes were made during and after the interviews to record non-verbal cues from participants. The interviews took place between July 2021 and December 2021 and lasted 28–47 min each (mean, 38 min). Data collection was discontinued when the methodological element of data saturation was achieved. Trustworthiness in terms of credibility was maintained by discussing the content of the study through continuous communication between the researchers and supervisor (PV).

#### Data analysis

Thematic content analysis was used to analyze the qualitative interview data (33). We placed considerable emphasis on demonstrating qualitative reliability (34). All authors independently and carefully reviewed and repeatedly read the transcripts to familiarize themselves with the narrative interview data and gain a better understanding and sense of them (33). The interview data were then categorized thematically. To identify quotations related to our research questions, PV created the initial codes from the interview data, and in the next step, suggested initial overarching themes. This study used both inductive and deductive coding methods. To further secure the analysis, the entire coding process was aided and organized using computer-assisted qualitative data analysis software (CAQDAS) (NVIVO, 2015). All researchers constantly checked whether the codes were used consistently (35). Initial codes were classified into more abstract and core categories using a constant comparative approach. Data analysis helped us generate several themes and subthemes. Next, the transcripts were reread to ensure that our themes were representative of the dataset. Each researcher engaged with other researchers to limit research bias and achieve effective communication and coordination. PV selected and drafted the initial illustrative quote. All other authors revised and suggested additional quotes. Researchers put all their efforts to limit unintentional personal bias.

## Results

The thematic data analysis revealed eight major themes and subthemes (Table 2).

**Table 2:** Major themes and subthemes.

**Theme**	**Subtheme**
1. Dying in isolation is an extremely inhuman experience, which COVID patients and their loved ones had to go through	1.1 Hospitalized critical COVID-19 patients are going through horror experiences
	1.2 Covid patients and family/friends have strong desire to see each other and get together
	1.3 Burying your loved one unclothed without saying goodbye is and intensely traumatic and stressful event
2. Epidemiology, liability, and proper conduct of nursing performance might provide support to strict visitation restrictions	
3. Remote communication technology should be available to every hospitalized COVID patient	
4. Physicians' and nursing practitioners' discretion and benevolent goodwill can mitigate the problem of dying alone	4.1. “Clandestine” visitations are allowed at physicians' discretion
	4.2. Physicians can make the process of dying in isolation milder at their discretion
	4.3. Nursing practitioners can make the process of dying in isolation milder at their discretion 4.4. Visitations should be allowed on an individual basis
5. Nursing practitioners' perception of nursing care expands to include holistic (biopsychosocial) care	5.1. Nursing practitioners show high levels of empathy toward family
6. Nursing practitioners experience intense psychological distress	6.1. Secondary post-traumatic stress and frustration
	6.2. Moral distress
	6.3. Feelings of demoralization
7. Health policy shifts away from the patient-centered care model	

### Dying in isolation is an extremely inhuman experience that COVID patients and their loved ones had to go through

#### Hospitalized critical COVID-19 patients are going through horror experiences

All participants emphasized that dying or nearing death in isolation due to the particular conditions of the COVID-19 pandemic is an unprecedented inhuman, unbearable, and devastating experience. “*COVID-19 disease is the disease of loneliness…*” (P9). Dying alone, without family members being allowed to say goodbye before, during, and after death, is an “*extremely undignified situation*” (P14). It is a “*soul-destroying*” situation (P2, P11). Participants said that caring for patients dying in isolation was the hardest [painful] piece of their work on COVID-19. (P5, P11). It is “*worse than the [COVID-19] disease itself* ” (P11). They repeatedly said that it is “*extremely inhuman*” (i.e., P7) or “*absolute horror*” (P1, P5). Participant P2 said, “*it is like to be going to be executed by a firing squad…*.” *Two* participants said that they thought that patients could go crazy in a COVID ward or ICU because of loneliness (P3, P14).

Patients who were staying in a COVID-19 ward were reported to experience psychological distress, characterized by a strong fear of death and loneliness. They are in an unknown (P7) and repulsive environment (P1) with monotonous sounds [beeping of monitors] (P1), in which patients constantly look at the sky. Patients are coping with the terrible feelings of being isolated from others and being totally dependent on others (P7) at the same time. They had infrequent and short interactions with “space-dressed” healthcare providers in a quasi-depersonalized environment (P9). In addition, they experienced intense physical distress at the same time (i.e., dyspnea). Participants stressed that every COVID patient room should be equipped with a TV to animate COVID patients (P3, P4, P5, P15). Participants stressed that the patients' eyes offered a striking expression of a strong fear of death, especially before they were intubated (P4, P5). They knew that intubation is often the last effort to stave off death (P4, P5, and P6).

Ultimately, many participants stated that there was no association between patient age and fear of death (P2, P7, P8, P12, P13, P14). Some participants said that younger patients expressed greater fear of death (P3, P6, P11), with others considering that older patients expressed greater fear of death than younger patients who “*feel invincible*” (P5, P9).

#### COVID patients and their family/friends have a strong desire to see each other and get together

All participants stated that critically ill patients and their relatives had a great need to see their loved ones in person. This was the main recurring theme in the data analysis. The following quotation indicates the following situation:

*[The patients] were feeling helpless, so lonely and anxious…There were patients struggling to get up from the unit bed in order to jump through the hospital window and meet their loved ones. Outside [the COVID ward], a patient's son was crying his heart out and said, “I'm losing my mother.” He begged us to see her, just to tell her a two-word phrase never said before: “I love you.” The mother was pleading with us, but we had to say no… That is how we did end up here… Outside [the COVID ward], souls were torn to pieces… However, inside, we could keep our duties*. (P14).

Participants said that they felt a strong urge to see and interact with their loved ones immediately after extubation (P7 and P14). A message from their loved ones was sufficient to make the patients have open and bright eyes (P3). Note that there are still very strong family bonds in Greek society (P13).

Finally, and most importantly, Greek people's distrust of their loved ones' health care providers results in their desire to be near their hospitalized loved ones (P5). This attitude is rooted in the culture of Greek society (P5).

*[A patient's daughter said,] I plead with you…I want to go in [the patient room] to see my mother, I want to say her goodbye…and she was crying…crying… she said, “I will do a COVID test,” … I will get dressed up in the protective suit... I beg you to let me see her... only to see her... and then…when the patient died, she went crazy and he was absolutely right”* (P7). Not surprisingly, “*The relatives were mourning before death. They felt helpless: They were irritated … it is reasonable”* (P14). Furthermore, participants P4 and P8 said that relatives wanted to see their loved ones, even from a distance, at least for a little bit of time.

The relatives preferred to stay near their loved ones' rooms, even though they knew that they had no chance of entering the rooms. Some of them were experiencing “silent pain” (P1, P7, P14). Participant P14 said, *Do you know how powerful scream is that silence? These looks are unforgettable…*

Relatives believe that patients might feel abandoned. This caused them to feel guilt. *They [relatives] were asking us to do our utmost… They wanted to bring small things for them, such as baby wipes, and asked us to convey their regard and a message to their loved ones … by saying that they [patients] are not abandoned…* (P4).

#### Burying your loved one unclothed without saying goodbye is an intensely traumatic and stressful event

This recurring finding emerged from participants' narratives. The following quotations describe heartbreaking situations and are representative of this point:

*Naked bodies, in tragic conditions, in big warbags. That was all it was? Is it the last salutation? Is there a right to respect their dignity? Do we have respect for the dead? This was all it was?* (P14). Indeed, *every human is unique; he has traced a path on Earth, and the moment he leaves deserves dignity and respect* (P12). The participants P3, P7, and P10 were in the same vein.

Ultimately and most importantly, “…*burying the unclothed deceased body of our loved ones is not consistent with our culture*.” (P5)

### Epidemiology. Liability and proper nursing performance might provide support for strict visitation restrictions

Among these reasons, strict visitation rules included those related to (a) epidemiology (precautions to prevent the spread of COVID-19) (P15), and (b) liability fears (i.e., P2, P4).

A third reason emerged from our data analysis: (c) preventing hindering the performance of nursing duties. The presence of family members in patient rooms might hinder the daily performance of nursing duties (P13 and P15). Note, however, that at the same time, participants P13 and P15 underscored the positive consequences of family presence at the bedside because family members near the patient's bed might relieve nursing practitioners of their duties to provide holistic care for critically ill patients. Importantly, our data analysis revealed that the presence of family members in patient rooms may help rather than hinder patients' well-being. Furthermore, participants argued that (c) due to strong family bonds in Greek society, allowing one family member to enter the patient room would press healthcare providers to permit more family members to visit their hospitalized loved one (P2, P4, P5, P6). Moreover, (d) getting dressed in protective clothing upon entry into patient rooms is a difficult skill for family members to learn and requires the assistance of specially trained nursing staff, which, however, is in shortage (P4). Ultimately, one participant (P2) expressed concerns regarding whether some visitors should be permitted to enter the patient rooms for fear of fainting because they wore protective clothing.

### Visitations should be allowed on an individual basis

Most participants clearly asserted that enforcing strict and unexceptional visitation restrictions is the cause of inhuman conditions and is completely unacceptable (e.g., P1, P12, and P14). At least visitations should be allowed before the patient was intubated (P9 and P12). Therefore, many participants expressed the opinion that hospitals should have the discretion to allow visits on an individual basis. This is a recurring finding. Most participants wondered why, in extreme and existential situations, family members could not enter patient rooms under the necessary protective measures, just like the hospital staff does (P1, P14). Otherwise, “*feelings, goodwill, and benevolence are lacking…*.” (Participant P14).

Notwithstanding, participants (P4, P6, P12) stressed that in some cases the patient-family encounter can be extremely harmful to a critically ill patient with low oxygen saturation because of the strong emotional reaction that can cause the patient: One *patient, after a loved one had visited him, was crying a lot… all night* (Participant P4).

### Remote communication technology should be available to every hospitalized COVID patient

All participants underscored the value of effective patient-family remote communication: “*Technology is a consolation for the relatives to see their loved ones opening and closing their eyes, to show signs of being alive…*” (Participant P2).

Ideal remote communication between patients and their loved ones requires both technical equipment (e.g., sufficient tablets and/or iPads) and trained personnel. At least a landline fix or wireless telephone should be accessible to all patients hospitalized in a shared room, given that the already existing fixed telephones in the hospital rooms cannot address the remote communication needs of patients (P12, P15). Moreover, they emphasized the need for healthcare settings to be adequately staffed by frontline nursing practitioners to secure ideal remote communication between patients and their families (P10).

### Physicians' and nursing practitioners' discretion and benevolent goodwill can mitigate the problem of dying alone

#### “Clandestine” visitations are allowed at physicians' discretion

“Clandestine” visits at physicians' discretion on an individual basis (despite the strict visitation restrictions in Greece) was a recurring finding that emerged from participants' narratives (e.g., P1, P4, P12, P13, P14, and P15). One participant said that while physicians were willing to allow people of higher social status to enter the patient room in secret, he (P14), with a broken heart and perhaps experiencing moral distress in the sense of “constraint distress,” had no choice but to prohibit patient's close relatives from entering the patient rooms for a few minutes, despite the fact that their loved one was dying! Nevertheless, physicians that permit relatives to pay causal visits to their loved ones might be motivated by compassion and goodwill (benevolent interest); *if someone was in great need [to see the hospitalized loved one], the issue was handled by physicians…* (Participant P13) … *Sometimes physicians are more tolerant of letting someone [see their loved one who is hospitalized] if he or she was constantly and intensely begging to visit the patient* (Participant P4), especially in extreme and tragic situations involving dying patients (Participant P15).

#### Physicians can make the process of dying in isolation milder at their discretion

Our data analysis revealed that in addition to the physicians' *de facto* discretion to allow family members to enter COVID patient hospital rooms (on an individual basis), it was up to their discretion to lessen the negative effects of strict no-visitor rules on both patients and family members, as an act of benevolent goodwill and generosity (P4). Physicians could (a) offer more or less support to those patients who were unable to make the best use of remote communication technology (P1, P2, P4, P7), and (b) spend more or less time with their patients (in the patient rooms) or spend more or less time informing family members of their loved one's condition (P1, P2, P7, P11, P13). Experienced nursing practitioners (participants) were of the opinion that providing adequate information to patients' relatives can serve as distress relief and put them at ease (P4, P9). However, participants said that providing information was far from being an easy task in light of the current circumstances due to the COVID-19 pandemic: “*[physicians] have to deal with a lot of people, they are dressed up, they cannot provide information to relatives*” (P10). Physicians may have no time to inform their relatives because of their overwhelming workload (P7). In addition, physicians may be reluctant to provide adequate information about a patient's condition because a COVID patient may get worse very quickly in an unforeseen way and relatives cannot understand this (P10).

Regarding the physicians' role in making dying in isolation milder at their discretion, the participants in this study highlighted the provision of information to family members rather than facilitating patients to utilize remote communication.

#### Nursing practitioners can make the process of dying in isolation milder at their discretion

The effective use of remote communication technology was not only at the discretion of physicians (as mentioned above) but also (and especially) at the discretion of nursing practitioners (P9, P13, and P14).

All participants highlighted the role of remote communication technology in reducing loneliness among hospitalized patients and psychological distress among family members. Participants used many words to describe their efforts to facilitate the use of remote communication technology at their discretion (voluntarily, as a gesture of goodwill), especially for the sake of patients unable to utilize it, to mitigate the negative consequences of strict no-visit rules.

For instance, participant P10 said, “…* I am dressed up [in the special protective suit] for a long time [and that makes me feel uncomfortable], I am sweating…[so]…. I have no time to open the camera*.” Whereas participant P11 said she often put a phone to the patient's ear, she never brought her own phone into a COVID patient room, with participant P12 saying that she brought her own phone in a patient room, enveloped with celluloid. To this effect, note that participants hesitated to come close to COVID patients due to their inner fear of contagion, including worries about being carriers of the COVID-19 virus and infecting their loved ones (P1, P4, P5, P7).

Furthermore, participants said that in addition to using remote communication tools, they were trying (at their discretion) various strategies to mitigate the negative consequences of strict no-visit rules. They said that they were “*trying different things to alleviate their pain [relatives' and patients' due to the ban on family visits to COVID patients]…*” (P13). Among these strategies were: (a) hospitalizing more patients (especially those who are relatives or around the same age) in shared hospital rooms to enjoy the company and mitigate isolation (P9, P13) while taking measures to protect their privacy and dignity, (b) being more hours near the patients and providing holistic care (including psychological support, see below) (P9, P13); (c) serving as mediators between patients and their family members (P3, P5), (d) allowing family members to bring small things for patients from outside, or even (e) looking the other way when family members take a sneak peek at their hospitalized loved ones, especially when the hospital's spatial setup makes it easy (P9).

Last, some nursing practitioners confessed that they made use of their *de facto* discretionary power to perform proper and dignified management of the deceased body (P10, P11): *I decided to dress them up… for that purpose, I was asking for an orderly's help… I did not want them to go naked in a bag… I did not want…* (P10). While physicians could allow visits at their *de facto* discretion (on an individual basis) despite the restrictions, nursing practitioners could perform proper and dignified management of the deceased body at their *de facto* discretion despite the protocols.

### Nursing practitioners' perception of nursing care expands to include holistic (biopsychosocial) care

During the COVID-19 pandemic, nursing care expanded to include additional layers of support and holistic care for critically ill patients (e.g., P4). Many participants highlighted the importance of involving themselves in providing holistic (multidimensional/bio-psycho-social) nursing care, aiming to address the needs of dying patients and families for psychological and spiritual support. They were willing to spend as much time as possible on their patents. They intended to hold the patient's hand, have proper verbal and non-verbal communication with them, provide psychological support, and play the role of relative or even confessor.

Many participants had a strong empathy-driven willingness to provide holistic and personalized nursing care. Participants clearly suggested that the unprecedented current pandemic circumstances caused them to instantly envision themselves and their family members in the patient's place. Many participants (e.g., P1, P4, P6, and P7). In addition to their basic nursing duties, they made great efforts, on the basis of benevolent goodwill, to stay closer to the patients to make the use of remote communication technology easier for them (sometimes using their own iPads) or provide them with holistic (bio-psych-social) support. The following quotation illustrates this point.

Furthermore, many participants said that they were playing the role of a psychologist (P5, P6, P9, P10, P11, and P15) or a confessor who provided spiritual support (P5). They felt obliged to facilitate conversations regarding patients' needs and wishes. At any rate, they attempted to play the roles of family members (P5, P7, and P15). Note, however, that nursing practitioners stressed that while they could feel the patient, they could not completely substitute the patient's family members (P8, P15).

The following are typical comments indicative of participants' strong willingness to provide holistic and personalized care for critically ill patients.

“*Nursing responsibilities include, but* are not limited *to, performing basic nursing care… [a nursing practitioner] have to go deeper into patient's inner world to meet his needs as much as you can… You have to build a warm relationship with your patient and play the role of a relative or a confessor…while you [a nursing professional] want to take off the protective suit that makes you feel uncomfortable, and get out of the patient room because you are afraid of the virus, you stay there because your work is so humanitarian…*” (P5).

“*OK…we build resilience over time; however, we make every effort to approach our work from a humanitarian perspective. We do the best, not only for patients but also for the sake of our soul…of course…*” (P1).

Interestingly, Participant P6 highlighted the value of achieving effective non-verbal communication in providing holistic and personalized care and said, “…*While performing routine nursing care, body language plays a crucial role in the interaction between patients and nursing practitioners. The way you [a nursing practitioner] enters a patient room…talk to the patient…look at him…touch him… Everything has a role to play [in providing holistic nursing care]..*. Participants placed great value on their facial expressions because *the only thing the patient can see when a nursing practitioner enters a COVID patient room are two eyes…*” (P10).

However, most importantly, given the striking shortage of the healthcare workforce, providing holistic nursing care would leave other patients without timely and proper nursing care. Participants' concerns about neglecting other COVID patients emerged as factors that constrained participants from abstaining from providing holistic care. To this effect, participant P12 complained that she had no time to cleanse the patient's body. Participants P7 and P9 said that they tried many times to spend time in the patient room holding their hands. However, the other patients did not receive timely nursing care. Participant P2 said, *I want to provide psychological support… but I cannot …I am forced to set priorities…I do my best so that the patient can survive…* In the same vein were participants P4, P14, and P15.

Finally, many participants insisted that a psychologist should regularly visit the COVID wards and provide support to COVID-19 patients (P5, P9, P11).

#### Nursing practitioners show high levels of empathy toward family

Many participants (i.e., P1, P4, P7, P12, P15) expressed strong empathy for what patients' relatives were going through: *We often put ourselves in relatives' shoes, and therefore, we often justified relatives' behavior* (P15). Participants emphasized that given that patients' symptoms could quickly turn serious and family members had no opportunity to follow the course of the disease, the process of putting a deceased body in an unclothed plastic bag was regarded by participants as extremely cruel, inhuman, undignified, and disrespectful; the dramatic reactions of the family members were completely reasonable (P12).

### Nursing practitioners experience intense psychological distress

#### Secondary post-traumatic stress and frustration

Providing nursing care for critically ill COVID-19 patients was reported as a traumatic experience that caused nursing practitioners to experience high levels of empathy-driven psychological distress. Witnessing the process of saying goodbye via remote communication technology before the patient becomes intubated was referred to as a highly stressful event: …*we were in patient rooms and have been witnessing that event…these were the most tough and sorrowful situations we had to deal with…* (P9). This is a recurring finding.

Traumatic events that occur in the workplace have serious implications for nursing practitioners' private lives (daily living). The participants said their psychological distress affected their private/family life rather than their care performance. Some participants placed considerable emphasis on the impact of their psychological distress on their private and family life (P3, P5, P7, P12, P14, P15). Many participants said they developed coping mechanisms to help manage painful or difficult emotions and maintain their ability to continue providing high-quality nursing care (P1, P2, P3, P5, P9, and P13). However, this is a difficult task. Participant P9 said that too much self-reflection was needed to maintain her emotional stability and ability to continue providing high-quality care for their patients. In a similar vein, Participant P7 said, “*We have experienced very traumatic events under tragic circumstances…which…will leave an indelible impression in our inner world…*.”

Participants' interviews suggested that the work-related traumatic scenes that they had experienced or witnessed at the workplace were internalized. The effective color of these internalized situations was extremely unpleasant and caused them to feel mentally and physically sluggish or unhealthy. “*I was a ‘going to work and coming home from work’ machine…I had abandoned everything in search of being able to remain strong enough in my job-related activities*” (P3). Participants described dramatic symptoms, such as insomnia, anxiety, depression, anger, and nervousness, negatively affecting their well-being (e.g., P7). Moreover, they confessed that trauma stayed with them long after the stressful event.

Participants could not get rid of thoughts, imageries, auditory hallucinations, and other post-traumatic symptoms, even while they were sleeping. “…*I kept hearing in my dreams beeping of patient monitors and asystole alarm sounds waking me up in the middle of the night…*.” (P 12). “*Thoughts and images were causing me to wake up in the middle of the night…absolute horror…This is such a terrible nightmare…*” (P14).

Furthermore, one participant's comment emphasized that nursing practitioners felt frustrated when they realized that all their efforts were in vain, namely, there was a high mortality rate. “…*you wonder what was the meaning of your effort?... You cannot get over it…*” (Participant P9).

#### Moral distress

As time-sapping overwhelming workload, further exaggerated by a striking lack of workforce (e.g., P2, P3, P4, P5, P6, P7, P9, P10, P12, P14, P15) prevented participants from providing holistic care, the perceived decline in quality of care triggered moral distress. All participants felt prevented from acting on what they knew to be the right, namely, spending time with patients to provide holistic care and facilitate communication between patients and family members, spending time with family members to support them, and providing proper care for deceased bodies, thereby compromising their moral integrity. These feelings challenged the participants' values/moral integrity. Many quotations cited elsewhere in the results section illustrate this point. Similarly, nursing practitioners' inner fear of contagion (mentioned elsewhere in this study), including concerns about being carriers of the COVID-19 virus and infecting their loved ones, acted as an inner constraint that caused them to experience moral distress (constraint distress).

#### Feelings of demoralization

Participants felt demoralized by being kept out of decisions about the care of COVID-19 patients despite the fact that they spent most of their time on frontline nursing activities, especially hands-on tasks. Most of them complained strongly about themselves being demoralized (P5, P7, P8, P9, P10, P11, P12, P13, and P14). They said that while they are the backbone, heart, and soul of the health care system (and spend a lot of time with patients), they are working in a physician-driven system (P8, P9) where neither policymakers nor physicians lend an ear to their voice.

Participants reported working like a robot, such as a machine-like humans, regarded as operatives of the care team (P7). They felt treated as an inferior part of the patient's care team because of hierarchies within interprofessional relationships. They said that the unprecedented circumstances of nursing during the COVID-19 pandemic shifted the balance of power in favor of physicians. Participants mentioned that physicians spent much less time in COVID patient rooms (P7) and felt that their voice was not heard by physicians (e.g., P14).

Despite the strict visitation restrictions imposed by the law, participants described physicians as being able to allow secret visitations to close family members in extremely exceptional cases. However, the participants and their colleagues could not do something like that. This made them feel inferior to the physicians.

### Health policy shifts away from the patient-centered care model

Political neglect has been mentioned as a major factor that increases the likelihood of dying in isolation. Adopting the patient-centered model of care entails facilitating effective in-person (on an individual basis) or at least effective remote communication between COVID-19 patients and families and promoting the provision of effective psychological support to COVID-19 patients. Participants were of the opinion that strict and unexceptional visitation restrictions are not consistent with *patient-centered and empathetic medicine*, which shows respect for humanity and fundamental human rights.

More specifically, the participants repeatedly highlighted the lack of a trained healthcare workforce. They said that the pre-existing lack of a healthcare workforce due to the recent financial crisis has been enlarged by the fact that many skilled but unvaccinated healthcare providers have been put out of the job by law. Furthermore, participants said that politicians passed the buck for crisis decisions to unvaccinated citizens and, more particularly, unvaccinated healthcare professionals, with the primary public healthcare sector, having received inadequate support from authorities despite the increasing influx of COVID patients (P7, P15): *[During the COVID-19 pandemic], the working conditions do not allow you [a nursing practitioner] to be human anymore*… *[…in the public health policy] there has been a recent shift from struggling to provide patient-oriented medicine toward passing the buck for crisis decisions to unvaccinated citizens and, more particularly, unvaccinated healthcare professionals, who have been put out of the job by law despite the (already in effect) lack of workforce in the healthcare sector. The authorities pass their responsibility to support the provision of patient-centered medicine to unvaccinated citizens…* (P7). Many participants highlighted the lack of workforce (P2, P3, P4, P5, P6, P7, P9, P10, P12, P14, and P15). The following quotation is indicative. “*We need additional staff members so as to have enough time to provide holistic care to these [critically ill] patients I have been trying to protest about it [these circumstances] … No answer! The only thing I hear all the time is ‘the virus is spreading fastest’…*” (Participant P14).

## Discussion

The testimonies collected in this study confirmed that dying a lonely death is very inhuman and far from being a “good death.” According to the British Geriatrics Society [Appendix in (25)] among other aspects of a good death comprised (a) having “control over who is present and who shares the end,” and (b) having time to say goodbye (namely, having the family present) (36). Dying without someone has considerable social and existential consequences for both patients and families (). It is argued that dying accompanied and having time to say goodbye (before, during, and after death) is a core element of what constitutes “good death” (37–40). However, it remains questionable whether “good death” is related to dying alone or accompanied (40, 41). A crucial point is the difference between existential loneliness and social (social) loneliness (42). Death is more than an individual's experience of death. This phenomenon belongs to the community (39). Dying alone is not justifiable (43). Lalani et al. put it best by saying, “Dying is a collective rather than an individual experience, and thus demands a collective approach to understand and respond to the needs of dying individuals and their families” (44). Furthermore, it is important to bear in mind that while putting vid patients in isolation is a radical precautionary measure to help prevent the spread of coronavirus disease (COVID-19), the impact of this measure on the course of the pandemic remains questionable (43). The dying communicating with and accompanied by family is said to be “ethically important” and beneficial for patients, family, and health professionals (4, 43). During the COVID-19 pandemic, the opportunity to say in-person a final goodbye and perform mourning rituals have been stolen from families (45, 46). These circumstances further complicate the process of accepting death and increase the psychological distress and emotional trauma of family members and friends (44, 45, 47–49), who feel complicated grief and express frustration (49). These circumstances reinforce “the painful nature of death” and prevent family members and friends from experiencing a “healthy mourning process” (45) healthy mourning process. On the contrary, persons who are “peacefully aware” (cognitively and emotionally) may experience lower levels of grief (50).

The testimonies collected in this study confirmed that dying in isolation, with family members being restricted from saying goodbye to their loved ones dying of coronavirus, before, during, or after death, is too inhuman to continue to be tolerated. As it is cruel and inhuman to die a lonely death, which, however, has become the “new normal” in the context of the COVID-19 pandemic, “administrative decisions regarding visitation policies must be critically examined and evaluated” (2). Scholars have strongly criticized strict visitation policies. Importantly, it has been argued that “such policies ‘prioritize, above all else, containment of the coronavirus,’ without considering other public goods, such as compassionate, family-centered care, reduction of fear, and improved health outcomes” (2). Relaxation of the general strict no-visitor rules should be decided on a case-by-case basis as much as possible (51, 52). Participants in this study were of the opinion that *a vague* policy should be enforced in Greece by enforcing a general no-visitation rule, which, however, allows visitors on an individual basis. Selman et al. found that infection control restrictions varied across regions and institutions (53). In Spain, death alone during the COVID-19 pandemic results from prevention measures imposed by protocols enacted by authorities (39). In the US, despite the fact that strict no-visitor rules have been considered “unavoidable reality,” necessary in service of the “greater good” (54, 55), the hospitals and other institutions caring for COVID-19 patients were considered the best decision-makers given that they had the knowledge and expertise to assess the particular situation (2). Many healthcare settings have enforced *strict* no-visitation rules during the COVID-19 pandemic, even during end-of-life circumstances. Other healthcare settings enforced *vague* policies by enforcing general no-visitation rules without clearly stating their guidelines and included a list of exceptions decided on a case-by-case basis. Furthermore, other health settings enforced a *lenient* visitation policy that allowed restricted and monitored visits to COVID-19 patients (2). Sudai advocates “for redistributing hospitals' discretion so that it is shared among additional stakeholders” in the USA (2). However, it should be highlighted that it is not easy to state clear guidelines regarding the visitation rights of hospitalized COVID-19 patients (56).

Furthermore, participants in our study reported (“illegal”) relaxation of strict no-visitor rules at the physicians' discretion. These sporadic instances can give rise to multiple types of inequalities.

Last, it is to be noted that authorities or hospitals should consider facilitating contact between patients and family members before starting the process of dying. Once the process of dying begins, the contact may no longer be meaningful because of the mental status of the patient.

Moreover, the participants highlighted the value of communication between nursing practitioners and family members. Initiating and holding end-of-life discussions with dying patients' relatives is negatively affected by the COVID-19 pandemic (37). However, such discussions are associated with better bereavement of relatives (6, 50). Ongoing and consistent communication between healthcare professionals and families is of paramount importance for providing patient-family-centered care in light of the unprecedented circumstances of the COVID-19 pandemic (6). Bio-psychosocial support should be provided not only before death but also afterward to address family members' bereavement and grief (6, 50).

All the participants in our study highlighted the need to use remote communication technology as a substitute for in-person contact between patients and their families or friends. However, while it is argued that meaningful communication can occur through the ideal use of remote communication technology (1, 57), the analysis of the testimonies collected in this study gives us the impression that the use of remote communication technology is not a substitute for in-person contact in the COVID-19 healthcare context. Studies have found that remote communication is an inadequate substitute for in-person contact between patients and family (44, 53). To this effect, Chen states “Virtual mourning, which is a novel, surreal experience has become a ‘new normality’ during the pandemic” (47, 48). However, nursing staff must be available to facilitate effective remote communication between family members and patients unable to use remote communication tools on their own (1).

Moreover, in the case of a patient dying in isolation, family members and friends may feel sadness, despair, hopelessness, frustration, anger, and guilt because they were not allowed to provide care and support for their loved ones when their loved ones needed them the most (6, 10, 53) and the fact that “the beloved relative or friend may feel terror or fear in dying alone” (10). During the COVID-19 pandemic, studies have shown “exceptionally difficult” experiences associated with bereavement (58). “Bereavement due to COVID-19 is characterized by a unique set of loss characteristics and circumstances and elevated grief levels” (49). British studies underscore family members' strong need to visit dying patients despite strict no-visitor rules (59–61). The findings that emerged from our data analysis reinforce previous literature.

Moreover, our finding that fear of virus transmission and fear of liability exposure might be the main reasons behind enforcing strict visitation restrictions (among other things) is in line with the findings of other studies (2). In addition, there is insufficient evidence to support the assumption that strict visitation policies reduce liability exposure or prevent virus transmission inside the hospital walls where “rigorous infection control measures” are implemented (2). It is arguably stated that enforcing strict visitation restrictions is facilitated by a cultural background where death has been gradually medicalized and dying in isolation is already long-established normality (2).

Participants in this study were strongly willing to provide holistic care (biopsychosocial care and substitute for their family members), highlighting physical touch and non-verbal communication (including facial expressions). Indeed, “good death” requires holistic care. Given the truth of the assumption that “emotional engagement in the contemporary clinical encounter” “may be used instrumentally to smooth the physician's work,” it is even more true when it comes to the health provider's work with vulnerable patients in critical condition (62). Cheng and Li Ping Wah-Pun Sin put it best by saying, “Providing holistic palliative and end-of-life care has been an integral part of our role in facilitating a good death” (63). To this effect, it is argued that “physical touch and non-verbal communication, such as facial expressions,” are “powerful tools to comfort and provide emotional support to patients and their caregivers” (44, 63). The British Secretary of State for Health and Social Care highlights the need to support the greater personalization of end-of-life care and address the spiritual needs of dying people (64). According to the British Geriatrics Society [Appendix in (25)], among other aspects of a good death, comprises access to spiritual and emotional support (36). Galbadage et al. outlined “an integrative approach to address the unique and holistic needs of critically ill patients dying with COVID-19” (65). The authors proposed a biopsychosocial model to address the biopsychosocial and spiritual needs of patients dying from COVID-19. Interestingly, the finding that the limited physical presence of family during the end of life increases existential and spiritual distress is mentioned in studies conducted in different cultures, such as in small towns and rural communities in Indiana, the United States, and Pakistan (44).

Importantly, as argued in the literature, providing holistic nursing care for dying patients is not easy. While emotional engagement is essential for clinical empathy, it may lead to chronic wearing and compassion fatigue. Therefore, emotional labor is vital for successfully performing such tasks (62). In addition, health providers cannot substitute for the offer of a family member or a beloved one, even though the involved health providers do their utmost to accompany dying patients and thus dignify death (39). This was included in the findings of the present study. Furthermore, empathetic communication between health professionals and critically ill patients is not easily attainable given that health professionals cannot spend adequate time with the patients mainly due to overwhelming workload, and the fact that they were “spaceship-dressed,” could only speak behind their shields (and other gears) and kept their own social distancing with the patients (43, 44). Although it is a difficult task, providing compassionate care for dying patients and families is a fundamental human right (66). Compassionate care for dying patients requires the presence of a person (health provider, if not a family member) near the dying patient. In the USA and Europe, the right “not to die alone” has been officially outlined by the Declaration on the Promotion of Patients' Rights in Europe (“Patients have the right to humane terminal care and to die in dignity”) (67), the Dying Person's Bill of Rights (“I have the right to not die alone”) (68), and the British Secretary of State for Health (64). The book “The rights of the dying patient,” written by Agius, encompasses many types of rights related to the process of dying. The “Right to Support” is included among them. In Appendix 2 (p. 147), it is stated that “Dying should not be an event suffered in isolation…support for the dying patient should come from family members and other people close to the patient” (69).

Furthermore, nursing practitioners' intense responses to difficult situations involving COVID patients dying alone emerged from our data analysis as a recurring finding. Nursing practitioners indeed have unique experiences working in a COVID-19 ward and must overcome extreme, totally new, and unpredictable situations that they experience as challenging and uncertain (70). These experiences cause psychological distress, including frustration and post-traumatic stress symptoms, and have a profound negative impact on psychological health (71, 72). Studies argue that the lack of opportunities to say goodbye before and after death is a complicated situation that impacts not only family members but also health providers or someone else close to death, resulting in these persons experiencing psychological distress (44, 46). Lalani et al. conducted a qualitative empirical study where visitation restrictions in the context of COVID-19 emerged as “the hardest piece” of nursing practitioners' work on COVID-19 (44). Furthermore, the authors state: “Considering the increased workload, caring for critically ill and dying patients, and lack of staffing and other resources, nurses remain unable to address their grief” (44).

Furthermore, Xu et al. found that nursing practitioners feel frustrated due to the fact that while they do their utmost to treat COVID patients, their efforts were not adequately “rewarded” in terms of patient survival (72). In this study, some participants expressed similar feelings.

Most participants in our study felt constrained to act contrary to what they believed to be an appropriate care plan or proper respect for a dead body. In other words, they have high levels of moral distress (73). In line with the findings of other studies, participants felt constrained to work under pressure due to a perceived decline in quality of care (72). Moreover, they felt internally constrained to remain hesitant in spending a lot of time with patients because of their inner fear of contagion (72).

Participants in this study said they put a lot of effort into maintaining their ability to care for patients. They expressed concerns about the quality of the nursing care provided as they experienced psychological stress. This finding is in line with that of previous studies (72). Organizational strategies should be developed to enhance nursing practitioners' resilience (74).

Furthermore, participants in our study felt demoralized because they were excluded from medical decisions regarding the care of their patients. This finding is in line with those of previous studies (72).

Participants in our study explicitly referred to the shift toward a less patient-oriented model of care. Drawing on the thematic analysis of the interviews, we identified this shift. This shift has been highlighted in prior studies (75). Xu et al. reported that during the COVID-19 pandemic, the rights of patients have been neglected, and health care has been dehumanized because of the restrictions imposed by health care authorities (72). However, in humanitarian crises, decisions and judgments should be made in light of humanitarian ethics (75). We resist accepting as morally right the consideration that “we must understand that in our culture of choice, the death we experience is not always the death we would choose” (10).

The humanitarian crisis of the COVID-19 pandemic has come into tension with individual human rights for the sake of public health and safety. In the new scenario, the hierarchy of principles inspiring care provided to hospitalized patients has been completely redefined on the basis of horizontal rules that apply across all healthcare settings. This puts again the spotlight on the tension between individual and group rights in the healthcare context. Goldberg argues that “open deliberation in a democratic social order is best served by acknowledging the constraints of the inescapably politicized process of public health policymaking” (76). In that regard, Gordberg states that “processes of medicalization tend also to emphasize downstream, micro-level (biomedical) interventions to health problems experienced by entire communities.” In view of the results of this study we argue for an analysis that captures the complexity and tension between individual and group rights (77).

Importantly, political neglect (resulting in a striking lack of trained healthcare workforce, among other things) was repeatedly mentioned or suggested as a major reason for the problem of dying alone due to COVID-19 in Greece. A range of health policy-related factors are mentioned in the Introduction section. We highlight the considerable emphasis that health policy placed on vaccination campaigns rather than on substantially strengthening healthcare services against the increasing influx and particular needs of COVID patients. Note, however, that during the COVID-19 pandemic, “a sense of political neglect or mistreatment was frequently expressed” in many other countries (53).

Ultimately, and most importantly, our data analysis revealed a range of cultural factors that exaggerated the negative experiences related to the phenomenon of “dying in isolation due to COVID-19” in Greece. We have provided an explanation for this finding. Despite the fact that Greece fully adheres to liberal European values and actual North European or American principle-based medical ethics (principlism), Greek healthcare ethics are deeply rooted in the so-called “Mediterranean bioethics.” Aristotelean ethics of virtue, the sanctity of life, spirituality, and friendship based on trust are among the essential components of the so-called “Mediterranean bioethics,” which developed by the thought of great Greek philosophers, Hippocrates, and great Mediterranean religions (Judaism, Christianity, and Islam) (78).

### Implications for practice and policy

We advocate the transfer of discretionary power from the state to the hospitals. Hospitals should be granted authority to consider how best to balance safety precautions while maintaining in-person contact between critically ill patients and their families. Schloesser et al. put it best in saying “Staying connected with seriously ill and dying patients must be facilitated, allowing face-to-face (shared), contact whenever possible, and allowing decisions to be made on an individual basis. It should always be possible to visit dying persons” (66). We hope that the hospitals' discretion will be “shared among additional stakeholders,” especially healthcare workers. Moreover, the broader use of remote communication technology should be offered to promote effective communication between dying patients and family members. Ideally, technology might be connected to a camera installed inside each patient room so that patients in the ICU do not need to touch any screen or keyboard. Finally, health policymakers should consider Greek society's deep-rooted moral values.

### Limitations

First, the sample size is small. Second, as this study mostly involved nursing practitioners working in Athens, the results may not be readily transferable to all healthcare settings throughout the country. Indeed, given the truth of the results of a recent study conducted by Lytras and Tsiodras in Greece, dying alone while being hospitalized in regions of Greece away from the capital region of Attica can make things much worse (32). However, these results are applicable to a larger number of nursing practitioners in many other healthcare settings in the country. Third, participants were not asked to check the consistency between their intentions and the results of the researchers. This limits the reliability of the study in terms of its confirmability.

## Conclusion

The inability of family members to say a final goodbye before, during, or after death by performing proper mourning rituals is extremely inhuman and profoundly impacts the mental health status of patients, family members, and frontline nursing practitioners. Patients and their family members strongly desire to see each other. Epidemiology, liability, and routine nursing performance emerged as reasons for the enforced strict visitation restrictions. Participants emphasized that visitations should be allowed on an individual basis and highlighted the need for the effective use of remote communication technology, which, however, does not substitute for in-person contact. Importantly, physicians allowed (illegal) visits on an individual basis. Nursing practitioners had strong empathic attitudes toward both patients and their families and a strong willingness (and most of the participants did their utmost) to provide holistic (biopsychosocial) care and pay respect for dead bodies (sometimes despite the restrictions). However, they felt prevented from doing what they knew was right, thus experiencing moral distress. Witnessing the process of saying goodbye via remote communication technology before the patient is intubated, as well as other heartbreaking scenes with patients and/or family, causes nursing practitioners to experience intense psychological distress, which impacts their family life rather than their nursing performance. Ultimately, there was a shift from a patient-centered care model to a population-centered care model, thus violating fundamental human rights and core values of medical ethics. Furthermore, we identified a range of policy- and culture-related factors that exaggerate the negative consequences of dying alone of COVID-19. The participants emphasized a striking lack of healthcare workforce. In Greece, strong family bonds still exist. Greek culture places a considerable emphasis on mourning rituals. Greek people distrust the healthcare providers of their loved ones and want to be present in the patient room. Perhaps these findings are related to the so-called “Mediterranean bioethics.”

These results reinforce the existing literature on several fronts. However, we identified some nuances related to political decisions and, most importantly, convictions that are deeply rooted in Greek culture. These findings are of great importance in planning tailored interventions to mitigate the problem of interest and have implications for other similar national contexts.

## Data availability statement

The original contributions presented in the study are included in the article/supplementary material, further inquiries can be directed to the corresponding author.

## Ethics statement

The studies involving human participants were reviewed and approved by the Ethics Committee affiliated with Aristotle University of Thessaloniki, Faculty of Health Sciences, Department of Medicine (No: 9.482/ 22-6-2021). The patients/participants provided their written informed consent to participate in this study. Written informed consent was obtained from the individual(s) for the publication of any potentially identifiable images or data included in this article.

## Author contributions

PV was responsible for the study conception, data analysis, ethical analysis of the findings, writing of the paper, and reporting of the study. AT interacted with the participants and performed the interviews, transcriptions, translations, and initial analysis. MD, ET, and AKT assisted in data analysis and revision of the paper. All authors have read and approved the final manuscript. All authors contributed to the article and approved the submitted version.

## Conflict of interest

The authors declare that the research was conducted in the absence of any commercial or financial relationships that could be construed as a potential conflict of interest.

## Publisher's note

All claims expressed in this article are solely those of the authors and do not necessarily represent those of their affiliated organizations, or those of the publisher, the editors and the reviewers. Any product that may be evaluated in this article, or claim that may be made by its manufacturer, is not guaranteed or endorsed by the publisher.

## Abbreviations

ICU: Intensive Care Unit.
